# Synergism between a foldase and an unfoldase: reciprocal dependence between the thioredoxin-like activity of DnaJ and the polypeptide-unfolding activity of DnaK

**DOI:** 10.3389/fmolb.2014.00007

**Published:** 2014-07-31

**Authors:** Rayees U. H. Mattoo, America Farina Henriquez Cuendet, Sujatha Subanna, Andrija Finka, Smriti Priya, Sandeep K. Sharma, Pierre Goloubinoff

**Affiliations:** DBMV, Faculty of Biology and Medicine, University of LausanneLausanne, Switzerland

**Keywords:** Hsp70, chaperones, misfolding, unfolding, aggregation, thioredoxins, protein disulfide isomerase, cochaperones

## Abstract

The role of bacterial Hsp40, DnaJ, is to co-chaperone the binding of misfolded or alternatively folded proteins to bacterial Hsp70, DnaK, which is an ATP-fuelled unfolding chaperone. In addition to its DnaK targeting activity, DnaJ has a weak thiol-reductase activity. In between the substrate-binding domain and the J-domain anchor to DnaK, DnaJ has a unique domain with four conserved CXXC motives that bind two Zn^2+^ and partly contribute to polypeptide binding. Here, we deleted in DnaJ this Zn-binding domain, which is characteristic to type I but not of type II or III J-proteins. This caused a loss of the thiol-reductase activity and strongly reduced the ability of DnaJ to mediate the ATP- and DnaK-dependent unfolding/refolding of mildly oxidized misfolded polypeptides, an inhibition that was alleviated in the presence of thioredoxin or DTT. We suggest that in addition to their general ability to target misfolded polypeptide substrates to the Hsp70/Hsp110 chaperone machinery, Type I J-proteins carry an ancillary protein dithiol-isomerase function that can synergize the unfolding action of the chaperone, in the particular case of substrates that are further stabilized by non-native disulfide bonds. Whereas the unfoldase can remain ineffective without the transient untying of disulfide bonds by the foldase, the foldase can remain ineffective without the transient ATP-fuelled unfolding of wrong local structures by the unfoldase.

## Introduction

Anfinsen demonstrated that under optimal artificial conditions, the primary amino acid sequence of a polypeptide contains sufficient information to direct its spontaneous acquisition of a native functional three dimensional structure (Anfinsen, 1973). However, protein crowding, which in the cytoplasm of human cells may reach up to 200 mg/ml (Geiger et al., 2012; Finka and Goloubinoff, 2013), can interfere with the productive folding of *de novo* synthesized polypeptides to their native state. This may occur under mild oxidative stress, in particular with large multidomain polypeptides that are aggregation-prone, where wrongly aligned disulfide bonds may detrimentally stabilize misfolded conformations (Martin and Hartl, 1997; Ellis, 2001; Cabrita et al., 2010). When a labile protein is exposed to a mild stress, such as heat-shock, it may transiently expose hydrophobic segments to the aqueous environment and seek thermodynamic stability by forming non-native intra- and inter-molecular beta-sheets, leading to the stabilization of insoluble protein aggregates (Dobson, 2003; Natalello et al., 2013). The non-native hydrophobic interactions of misfolded proteins with membranes and other labile members of the proteome can have toxic effects, in particular for neurons. Membrane damage can induce the formation of reactive oxygen species (ROS) and trigger chronic neuro-inflammation and apoptosis, leading to tissue-loss and degenerative diseases, such as Alzheimer's, Parkinson's and aging in general (Bucciantini et al., 2002; Selkoe, 2004; Hinault et al., 2006). ROS particularly target unsaturated lipids and proteins with exposed reduced cysteines, leading to protein dysfunction, crosslinking and aggregation (Thomas and Mallis, 2001).

Prokaryotes and eukaryotes have evolved a complex network of molecular chaperones that in unstressed cells regulate the activity of alternatively- vs. natively-folded proteins such as clathrin cages vs. triskelions, and in stressed or diseases cells prevent and even actively avert the formation of cytotoxic misfolded protein conformers (Finka et al., 2011; De Los Rios and Goloubinoff, 2012). Hence, stable, potentially toxic, misfolded and aggregated polypeptide species can be actively converted by disaggregating and unfolding chaperones into harmless, natively refolded functional proteins (Parsell et al., 1994; Sharma et al., 2010; Hinault et al., 2011; Shorter, 2011; Rampelt et al., 2012; Mattoo et al., 2013). The mechanism of unfolding involves the initial binding to a chaperone surface of an otherwise stably misfolded polypeptide substrate with exposed hydrophobic surfaces. Following the ATP-fuelled unfolding and release, the unfolded intermediate may then spontaneously refold into its most stable native state and thus escape aggregation or chaperone re-binding (Sharma et al., 2010; Priya et al., 2013b). Similarly, the mechanism of folding by a protein disulfide isomerase involves the oxidoreduction-dependant transient covalent binding of a thiol-disulfide oxidoreductase to a misaligned cysteine pair that may wrongly stabilize a tensed misfolded structures in the polypeptide. The transient opening of the misaligned cysteines may allow a relaxation of the local tensions in the misfolded polypeptide structure, thereby permitting the proper repositioning of the cysteines in the native structure (Ou et al., 2001; Irvine et al., 2014).

Prokaryotic DnaK and its eukaryotic orthologous Hsp70 and Hsp110, are polypeptide unfolding molecular machines that can use the energy of ATP hydrolysis to unfold various polypeptides in different stable conformations (Sharma et al., 2010; Mattoo et al., 2013). DnaK uses DnaJ as a substrate-recognition co-chaperone that preferentially binds to misfolded (Hinault et al., 2010), or alternatively folded polypeptides (McCarty et al., 1996) with exposed hydrophobic residues, but avoid binding to compact natively-folded proteins with few exposed hydrophobic residues (Hinault et al., 2010). Thus, by way of its conserved J-domain that anchors to the nucleotide binding domain of DnaK in the ATP-bound, but not to the ADP-bound state, DnaJ may recruit a stably misfolded polypeptide to the unfolding machinery of DnaK, to be then released and refold to the native state (Sharma et al., 2010).

*Escherichia coli* DnaJ is a type I J-domain protein (J- protein) that, like human DNAJA1, has a conserved antiparallel beta-sheet domain with four characteristic CXXC motives, each being typical signatures of the thioredoxin catalytic site. Two Zn^2+^ ions can bind with high affinity to the two cysteines tetrads (Banecki et al., 1996). This compact cysteine-rich domain is flanked on one side by the conserved 8 kDa α-helical J-domain, responsible for anchoring to DnaK, and on the other the β-sheet protein-binding domain *per se*. This cysteine-rich domain, which is also called the zinc-finger domain, is absent in type II J-proteins, such as *E. coli* CbpA and human DNAJB1 (Ramos et al., 2008; Kampinga and Craig, 2010) and also from the less conserved type III J-proteins, such as *E. coli* DjlA and human auxilin. In the cell and also *in vitro*, the bacterial CbpA can substitute for DnaJ in DnaK-mediated polypeptide unfolding/refolding assays (Ueguchi et al., 1994; Bird et al., 2006; Hinault et al., 2010).

Thioredoxins are small ubiquitous redox enzymes that reduce protein disulfide bonds by using a pair of cysteine residues present in a strictly conserved WCGPC catalytic motif. Thioredoxin-2, has two WCGPC catalytic motives that can form a zinc-finger, similar to the Zinc (Zn) Center I and II to be found in DnaJ, each with four cysteines coordinated to a single Zn^2+^ (Ye et al., 2007). Zn Center I of DnaJ was reported to be essential for substrate binding and Zn Center II for its interaction with DnaK (Linke et al., 2003). In addition, DnaJ has also been shown to carry a weak thiol-reductase activity and it has been suggested to act as a protein disulfide isomerase (de Crouy-Chanel et al., 1995). Yet, the complete deletion of the cysteine rich domain of DnaJ did not have dramatic effects, both *in vivo* and *in vitro* ability of DnaJ to co-chaperone DnaK's activity (Wall et al., 1994; Banecki et al., 1996). This was confirmed in protein disaggregation and refolding assays using heat-denatured glucose-6-phosphate dehydrogenase (G6PDH) as substrate, where in the presence of 5 mM DTT, CbpA devoid of a Zinc-finger domain was almost an equally effective DnaK co-chaperone, as DnaJ that contained a Zinc-finger domain (Hinault et al., 2010).

Here, we re-examined the function of the cysteine-rich region of DnaJ, as a putative dithiol-oxidoreductase foldase, in the light of our recent findings that human Hsp70 and Hsp110, and also bacterial DnaK, can act as catalytic ATP-fuelled polypeptide unfoldases on various stable misfolded and aggregated protein substrates (Sharma et al., 2010; Mattoo et al., 2013). We created a truncated version of DnaJ (ΔDnaJ) lacking both Zn centers (residues 144–195). *In vitro* assays showed that the unfoldase activity of DnaK was optimal at refolding mildly oxidized substrates to the native state, when it was synergically assisted by externally added thioredoxin, or by the intrinsic thiol-reductase activity of wild type DnaJ. Reciprocally, the thiol-reductase activity of *E. coli* thioredoxin was effective at refolding mildly oxidized substrates only when it was synergically assisted by the ATP-dependant unfoldase activity of DnaK.

## Materials and methods

### Proteins

DnaK, DnaJ, and ΔDnaJ purification was according to Gur et al. (2004). GrpE was a gift from H.-J. Schönfeld, F. Hoffmann-La Roche, Basel, Switzerland. Glucose-6-phosphate Dehydrogenase (G6PDH) from *Leuconostoc mesenteroides*, Bovine rhodanese, Bovine Insulin and Thioredoxin from *Spirulina* sp. was purchased from Sigma-Aldrich. Protein concentrations were estimated by the Bradford Assay and protein concentrations were always expressed in protomer.

### Insulin turbidity assay

The insulin turbidity assay was performed as described in Holmgren (1979) with following modifications; Native insulin (0.13 mM) supplemented either with thioredoxin (1 μM and 10 μM), DnaJ (30 μM) or ΔDnaJ (30 μM) in presence of 0.45 mM DTT (Sigma-Aldrich). Light scattering of aggregates formed was measured in a fluorimeter at 650 nm.

### Chaperone refolding assays

Heat-preaggregated G6PDH was refolded by the DnaK chaperone system as described in Ben-Zvi et al. (2004), with the following modifications; 650 nM heat-aggregated G6PDH (final concentration) was reactivated in the presence of 5 μM DnaK, incrementing (0–3.5 μM) DnaJ/ΔDnaJ, 2 μM GrpE (the full DnaK chaperone system) and 5 mM ATP. G6PDH activity was measured at different times of chaperone-mediated refolding reaction at 30°C. Urea-denatured rhodanese was refolded by the DnaK chaperone system as described in Mendoza et al. (1991) with the following modifications; Urea inactivated rhodanese (500 nM) was refolded at 25°C, in the presence of limited amounts of DTT (0.1 mM), without or with DnaK (3 μM), DnaJ or ΔDnaJ (1.6 μM), GrpE (1 μM), ATP (5 mM) and with or without Thioredoxin (1 μM).

### Reconstitution of a typical DnaJ dimer from published structures of J-proteins

#### Method

**Figure 1:**
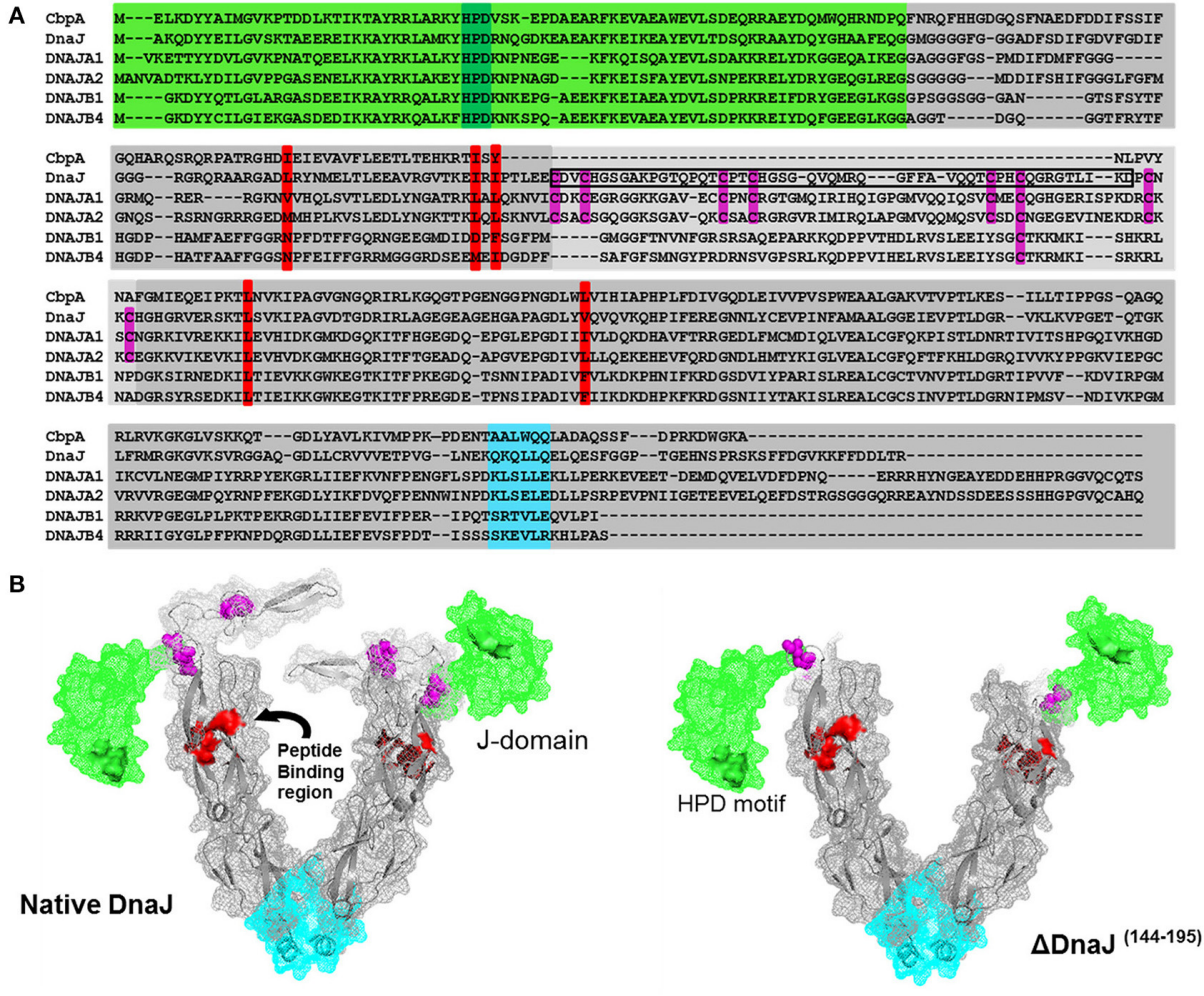
**Sequence alignment of the two most abundant human and bacterial J-proteins. (A)** Amino-acid sequence alignment of the two most abundant Hsp40-like J-proteins in *Escherichia coli* bacteria CbpA and DnaJ, and the four most abundant Hsp40s in human: the type I J-proteins DNAJA1 and DNAJA2 and type II J-proteins DNAJB1 and DNAJB4. The conserved N-terminal J-domain (light green) with the conserved HPD motif (deep green) is followed by a G/F rich hinge region. In DnaJ, DNAJA1 and DNAA2, this is followed by a Zinc binding domain (light gray) with four pairs of highly conserved cysteines (purple), which are absent in CbpA, or different in DNAJB1 and DNAJB4. This is followed by a C-terminal peptide-binding domain (PBD) (dark gray) with the residues involved in peptide binding groove shown in red and the putative dimerization interface shown in cyan. The black rectangular frame shows the 51 cysteine-rich residues, which we deleted from bacterial DnaJ that optimally match domain alignments with CbpA. **(B)** Left panel: Approximate 3D structure of a typical DnaJ dimer, reconstructed from sequence and structural alignments of various partial X-ray crystallographic structures from bacteria, yeast and human Hsp40s (PDB: 1XBL the *E. coli* J-domain; 1EXK for the *E. coli* cysteine rich domain; 1NLT for the yeast YDJ1 PBD+cysteine rich domain; 3AGZ for the human HDJ1 for half PDB+dimerization+two bound peptides; 2QLD for the human HDJ1 for Half PDB+dimerization). Right panel: The structure of mutant DnaJ (ΔDnaJ). The colored segments are as in **(A)**.

The various structures of J-proteins from Protein Data Bank (PDB) were aligned using the align function in the PyMOL program and were also sequence-aligned according to the multiple sequence alignment with hierarchical clustering (Corpet, 1988). The various missing structures in the *E. coli* DnaJ dimer were approximated from the overlapping structures from the closest sequence-relatives of DnaJ. The final structure in Figure 1B is composed of the J-domain from *E. coli* (PDB: 1BQ0), the cysteine-rich region and most of the C-terminal domain from yeast DnaJ, ydj1 (PDB:1NLT) and the dimerization interface was contributed by the human hdj1 (PDB: 3AGY), see also Supplementary Figure 1.

## Results

### Sequence and structural comparisons show that type II J-proteins lack a conserved cysteine-rich domain common to all type I J-proteins

Amino acid sequence alignment of DnaJ and CbpA, which are the main J-domain co-chaperones in *E. coli*, show a very high homology of their respective N-terminal J-domains (Figure 1A, Green background) and a good homology of their C-terminal protein-binding domains (Figure 1A, gray). Amidst the J-domain and protein-binding domain, DnaJ has a conserved Zn-binding domain with four characteristic CXXC pairs at conserved positions of the anti-parallel beta-sheet (Figure 1A, cysteines in purple). Whereas the *E. coli* CbpA sequence is devoid of cysteines and plainly lacks this domain, corresponding to residues 144–195 in DnaJ, these residues encode for most of the Zn-finger motives. Confirming this, the crystal structure of the *Thermus thermophilus* DnaJ_2_ dimer, which is typical CbpA ortholog, lacks cysteines and has no Zn-finger domain (Barends et al., 2013). Sequence alignments of *E. coli* DnaJ and CbpA with four of the most abundant J-domain proteins in the human cytoplasm (Finka and Goloubinoff, 2013), confirmed that human DNAJA1 and DNAJA2 contained the four characteristic CXXC pairs at conserved positions as in DnaJ, whereas DNAJB1 and DNAJB4 contained a slightly shorter middle domain lacking cysteines (Kampinga et al., 2009; Kampinga and Craig, 2010) (Figure 1A).

In an attempt to understand the particular function of the Zn-fingers in type I J-domain proteins, we produced an *E. coli* DnaJ mutant lacking residues 144–195. Alignments showed that the deletion precisely corresponded to the missing cysteine-rich segment in CbpA (Figure 1A). Noticeably, among the four conserved pairs, the most distal C-terminal CXXC pair has been shown to contribute to the binding of substrate polypeptides to DnaJ (Linke et al., 2003). As sequence alignments in various CbpAs and DnaJs suggested that this segment should be present but lack cysteines, our CbpA-mimetic ΔDnaJ was designed to still contain the most C-terminal cysteine pair out of the four pairs present in wild-type (WT) DnaJ.

We used partially resolved X-ray crystal structures from various orthologs of J- proteins from eukaryotic and prokaryotic type I and Type II J-proteins (see Materials and Methods), to generate a 3D reconstruction model of a typical *E. coli* DnaJ dimer (for 3D view of the DnaJ dimer, watch Movie 3DHsp40) (Figure 1B). Confirming the validity of our reconstruction, the model showed a very high structural homology with the near complete X-ray structure of the type II dimer of *Thermus thermophilus* DnaJ_2_, which is devoid of cysteines (Supplementary Figures 1A,B). In the model, the cysteine-rich domain (Figure 1B, purple cysteine pairs) appeared as an independent, compactly folded middle domain, without apparent close interactions with the flanking domains: It was merely connected at E143 to the N-terminal J-domain (Figure 1B, green), and at P196 to the C-terminal protein-binding domain (Figure 1B, gray background, peptide-binding residues in red). Because in the DnaJ structure positions for E143 and P196 are in close vicinity, the deletion of the 51 interspacing residues in ΔDnaJ was not expected to cause any significant structural tension or destabilization in the other parts of the molecule (Figure 1B right). Thus, the ΔDnaJ mutant was a structural mimetic of CbpA. We next used well-established stringent *in vitro* ATP-DnaK-dependant, GrpE-regulated protein unfolding/refolding refolding assays, to address the specific role of the cysteine-rich domain in Type I, as compared to Type II J-proteins cochaperones.

### Wild-type DnaJ but not ΔDnaJ can catalyze the reduction of insulin

**Figure 2:**
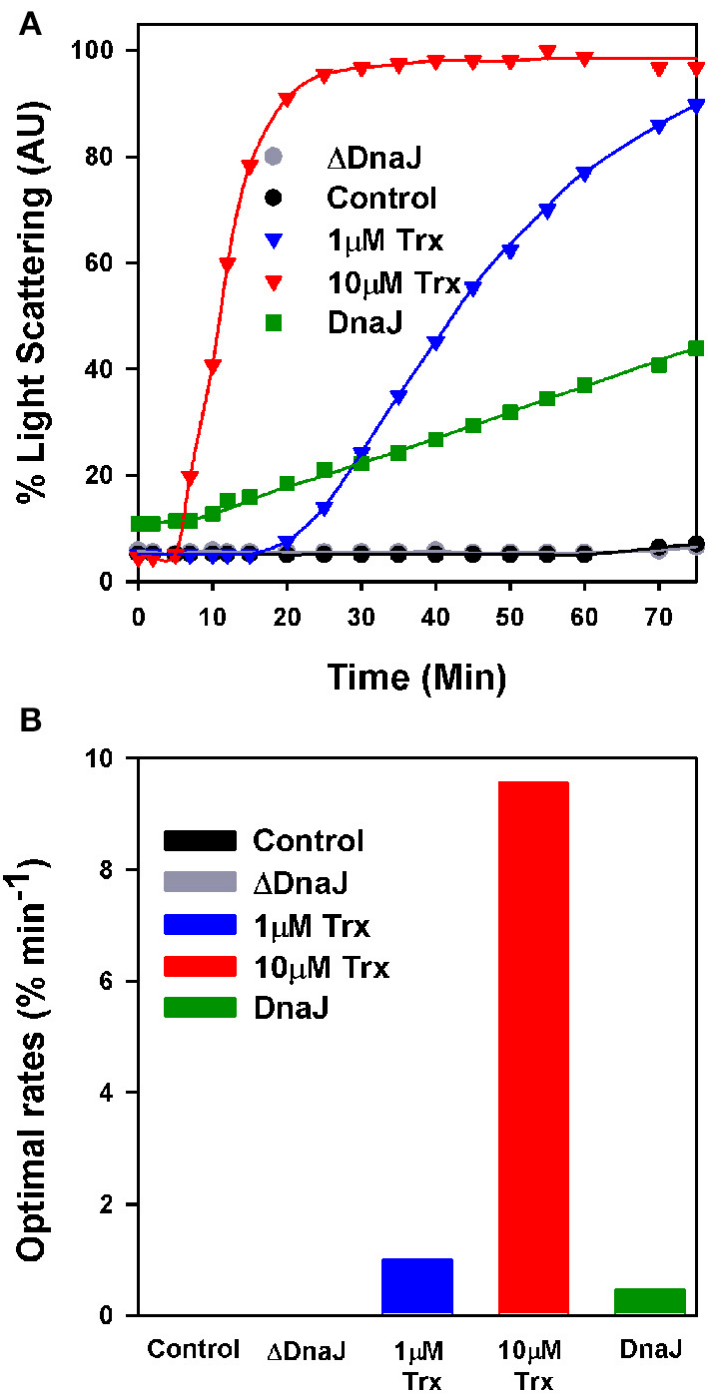
**The cysteine-rich domain of DnaJ has a thiol-reductase activity. (A)** Time dependent insulin turbidity assay: Native insulin (130 μM) in presence of 0.45 mM DTT was supplemented either with thioredoxin (1 and 10 μM), DnaJ (30 μM) or ΔDnaJ (30 μM) as indicated. The maximal insulin light scattering signal at 40 min following addition of 10 mM DTT was set as 100%. **(B)** Optimal rates of insulin turbidity formation calculated in the linear region, from the inflection point, of the sigmoidal curves of **(A)**.

When supplemented with 10 mM DTT, mature insulin readily forms large turbid aggregates that within minutes maximally scatter light at 650 nm (Holmgren, 1979). In contrast, in the presence of limiting DTT (0.45 mM), the very low light-scattering signal of native insulin did not increase during the first 60 min of the assay, indicating that within this time range, the low concentration of DTT was poorly effective at reducing insulin into aggregation-prone species that scatter light (Figure 2A). Expectedly, when in the presence of 0.45 mM DTT, 1 μM or 10 μM of *E. coli* thioredoxin were added, insulin turbidity rapidly increased at the respective rates of 1% min^−1^ and 10% min^−1^ (maximal velocities were measured at the inflection point of the sigmoidal curves) (Figure 2B). When instead of thioredoxin, WT DnaJ (30 μM) was added, insulin turbidity also steadily increased, yet at a slow rate of 0.4% min^−1^ (Figures 2A,B). In contrast, the same amount of ΔDnaJ remained ineffective at accelerating insulin reduction, demonstrating that the Zn-fingers domain of DnaJ carries a mild thiol-reductase activity.

#### ΔDnaJ functions as a bona fide DnaK targetase

**Figure 3:**
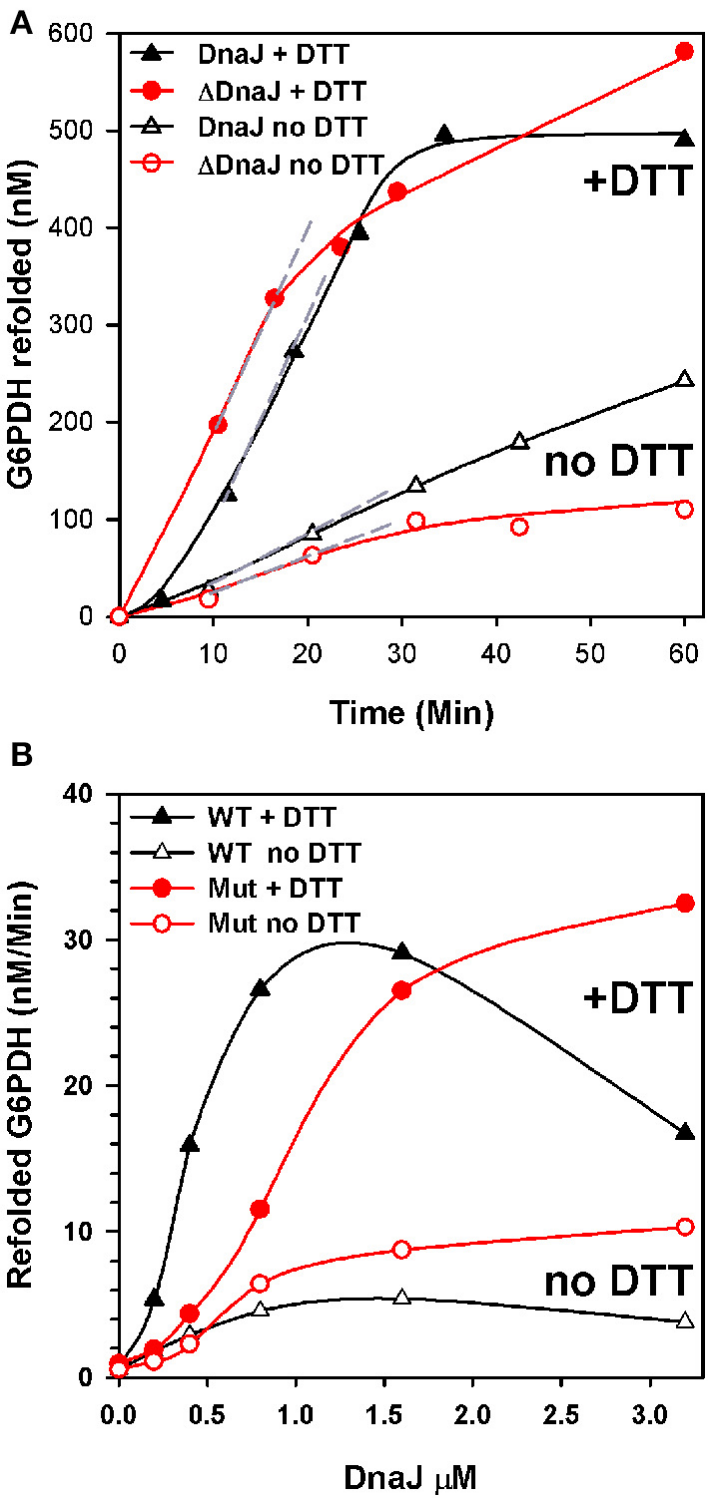
**ΔDnaJ is as effective a co-chaperone as DnaJ. (A)** Time-dependent refolding of heat denatured G6PDH. 0.65 μM G6PDH was first heat-denatured and then supplemented at 30°C with 5 μM DnaK, 2 μM GrpE and 1.6 μM DnaJ or 1.6 μM ΔDnaJ in absence (empty symbols) or presence (filled symbols) of 5 mM DTT. (**B**) Rates of G6PDH refolding obtained from the maximal slopes (shown as dotted gray lines in **A**) at different concentrations of DnaJ or ΔDnaJ, as indicated.

We next addressed the ability of ΔDnaJ, as compared to WT DnaJ, to target a stable pre-aggregated substrate onto the DnaK unfolding chaperone machinery. We used as model substrate, the cysteine-less G6PDH enzyme, which was first heat-preaggregated in the absence of chaperone (Diamant et al., 2000; Hinault et al., 2010) and subsequently supplemented with constant concentrations of DnaK, GrpE and ATP, and increasing concentrations of ΔDnaJ or DnaJ, as indicated (Figure 3). The time-dependent reactivation of G6PDH by DnaK, GrpE and ATP at 30°C in the presence of increasing amounts of ΔDnaJ or WT DnaJ (Figure 3A) provided maximal refolding rates, which were plotted against the J-co-chaperone concentrations (Figure 3B). The chaperone reactions were found to be driven equally well by 1.6 μM WT DnaJ as by 3.2 μM ΔDnaJ, at an apparent maximal refolding rate of 30 nM min^−1^. This demonstrates that under the fully reducing conditions (10 mM DTT) of the *in vitro* assay, the Zn-finger domain was not essential for the ATP-fuelled unfolding/refolding of stable pre-aggregated polypeptides by DnaK. This was not unexpected, as CbpA that naturally lacks a Zn-finger domain, has been shown earlier to carry a *bona fide* DnaJ-like (Mattoo and Goloubinoff, 2014) co-chaperoning activity *in vitro* (Gur et al., 2004; Hinault et al., 2010; Mattoo and Goloubinoff, 2014). Noticeably, 2.5 times more ΔDnaJ (1 μM) was needed to achieve half the maximal refolding rates of the reaction than WT DnaJ (0.4 μM). Thus, ΔDnaJ had an apparent lower affinity for the misfolded G6PDH substrate than WT DnaJ, similar to CbpA (Hinault et al., 2010) and in agreement with the earlier observations by Linke et al. (2003) that the Zn-binding domain participates, although is not essential to substrate binding.

When the reaction was performed without DTT, the ATP-fuelled DnaK-DnaJ-GrpE-mediated refolding of stably heat-denatured G6PDH was strongly reduced. Although this reduction can be attributed in part also to a possible oxidation of the single cysteine residue at position 15 of DnaK, the decrease of the chaperone performance was noticeably more pronounced with the cysteine-rich DnaJ than for the cysteine-deficient ΔDnaJ. Thus, without DTT, the activity of 1.6 μM DnaJ was 6 fold slower than with DTT, as compared to only 3 fold slower in the case of 1.6 μM ΔDnaJ (Figure 3B). This suggests that under non-reducing conditions, the highly reactive cysteines in type 1 J-co-chaperones may form various covalent bonds with other exposed cysteines in DnaJ and possibly with the single cystein of DnaK (but not with the substrate which is cysteine-less) that adversely affect substrate processing by DnaK.

### Thioredoxin potentiates the DnaK unfoldase activity and DnaK potentiates the thioredoxin foldase activity

**Figure 4:**
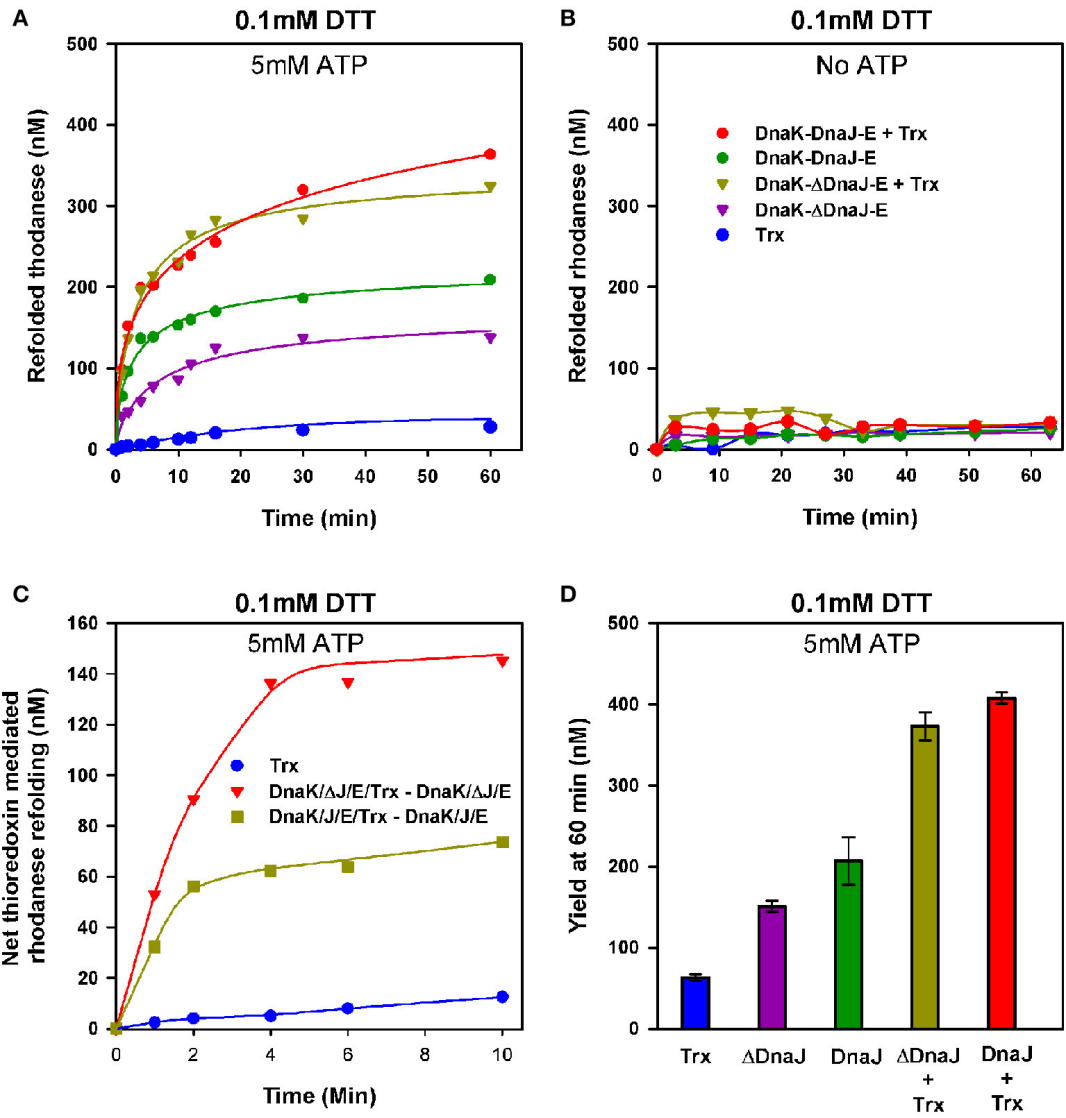
**Thioredoxin-1 enhances unfolding of oxidized polypeptide substrates by DnaK. (A)** Urea inactivated rhodanese (0.5 μM) was refolded at 25°C, under limited amounts of DTT (0.1 mM), without or with 3 μM DnaK, 1.6 μM DnaJ or ΔDnaJ, 1 μM GrpE and 5 mM ATP in absence or presence of 1 μM Thioredoxin as indicated. **(B)** Refolding of urea inactivated rhodanese under the same conditions as in **(A)**, but without ATP. **(C)** The net effect of added thioredoxin upon DnaJ or ΔDnaJ in DnaK-DnaJ-GrpE mediated refolding, from **(A)**. **D)** Yield of rhodanese refolded at 60′ under similar conditions as in **(A)**.

We next used urea- pre-denatured rhodanese, which at varience with G6PDH, contains four cysteines and is accordingly an oxidation-sensitive stringent protein substrate of DnaK (Natalello et al., 2013), to address the possible contribution of the thiol-disulfide oxidoreductase activity of DnaJ's Zn-finger domain to the ATP-fuelled unfolding/refolding activity of DnaK. Urea-pre-unfolded rhodanese was diluted in 0.1 mM DTT, without or with 1 μM of *E. coli* thioredoxin, ATP, DnaK, GrpE and DnaJ (KJE) or ΔDnaJ (KΔJE), as specified. With ATP, DnaK together with GrpE and DnaJ expectedly drove the native refolding of urea-denatured rhodanese at the maximal rate of 23.6 nM min^−1^. As in the case of DnaK-mediated refolding of heat-predenatured G6PDH, the rate was about twice faster with DnaJ than with ΔDnaJ (Figure 4A). Noticeably, thioredoxin (Trx1) alone or with KJE chaperones but without ATP, did not drive any significant spontaneous rhodanese refolding (Figure 4B). In contrast, with ATP, thioredoxin doubled the rates of the chaperone reaction. In the first 15 min of the reaction, the thioredoxin effect was more pronounced in the case of the ΔDnaJ- than of the DnaJ-mediated reaction (Figure 4C), suggesting that thioredoxin could complement the slow, yet significant effect of the cysteine-rich domain of the wild type DnaJ, which was completely lacking in ΔDnaJ. Thus, the unfolding action of the DnaK chaperone synergistically activated, remarkably in an ATP-dependent manner, the “foldase” activity of thioredoxin. Reciprocally, thioredoxin synergistically activated the ATP-driven unfolding/refolding activity of the DnaK chaperone, as the thioredoxin contribution was more effective with ΔDnaJ that lacked thioredoxin-like foldase activity, than with DnaJ that already carried an intrinsic thioredoxin-like activity of its own (Figures 4C,D).

## Discussion

By virtue of their conserved J-domain that specifically “locks” into the ATP-bound Hsp70 unfolding chaperone machines, J-proteins can specifically target various polypeptide substrates that need, at some point, to be unfolded in the cell (Mattoo and Goloubinoff, 2014). The binding of a J-domain to Hsp70 also triggers ATP hydrolysis, which in turn causes the tight closure of the lid onto the base of the protein-binding domain. This closure can apparently apply a local “clamping” destabilizing force onto an entrapped bulky secondary structure from the bound polypeptide substrate and entropically pull on it (De Los Rios et al., 2006; Baneyx and Nannenga, 2010; Sharma et al., 2010). Following ADP-accelerated release from Hsp70/Hsp110 by a nucleotide exchange factor (bacterial GrpE, or eukaryotic Bags), the locally unfolded polypeptides may then dissociate and spontaneously reach the stable native state with a low affinity for J-proteins and for the Hsp70/Hsp110 chaperones (Sharma et al., 2010; Priya et al., 2013a; Mattoo and Goloubinoff, 2014). Thus, auxilin is a J-protein that can target Hsc70 onto clathrin cages, to be locally pulled upon and deoligomerized into triskelions (Ma et al., 2002; Jiang et al., 2003). Zuotin is a J-protein that can target HSPA14 onto *de novo* synthesized polypeptides exiting the ribosome, to be pulled upon, unfolded and imported into the cytoplasm (Gautschi et al., 2001), Sec63 can target BIP onto partially folded cytoplasmic polypeptides, to be pulled upon, unfolded and imported into the ER lumen (Misselwitz et al., 1999) and PAM18:PAM16 targets mHsp70 onto partially folded cytoplasmic polypeptides to be pulled upon, unfolded and imported into the mitochondrial matrix (Pais et al., 2011). Similarly, following stress, human DNAJA1 or bacterial DnaJ and CbpA can target stable stress-misfolded and aggregated inactive proteins, respectively onto human Hsc70 and Hsp110, or bacterial DnaK, to be pulled upon, unfolded into natively refoldable proteins (De Los Rios et al., 2006; Mattoo et al., 2013; Priya et al., 2013a). Suggesting a catalytic action of the J-domain cochaperones, their copy number in the various compartments of the human cell is about one order of magnitude lower than the sum of their target chaperone partners (the cytoplasmic Hsp70, Hsc70 and Hsp110s, the ER BIP and Grp170 and mitochondrial mtHsp70) (Finka and Goloubinoff, 2013). Likewise, in log growth phase, the copy number of the *E. coli* J-proteins is about 6 times less than DnaK and HscA (Arike et al., 2012). Moreover, *in vitro* chaperone refolding assays showed that 20 times less DnaJ molecules compared to DnaK sufficed to drive the ATP-dependent stringent refolding of stable heat-denatured proteins at half maximal rate (Hinault et al., 2010). Together, this suggests that in each *E. coli* cell, 6000 molecules of DnaK unfoldases can be optimally put in relation with misfolded polypeptide substrates by merely 180 DnaJ dimers implying multiple iterative cycles of co-chaperone binding and release (Hinault et al., 2011; Arike et al., 2012).

On top of the well-established DnaK polypeptide targeting-for-unfolding function of DnaJ, we addressed here a particular thiol-disulfide oxidoreductase sub-function, which we found to be specifically associated to the cysteine-rich domain of DnaJ, as a characteristic of type I J-proteins in general. Under reducing conditions as in the unstressed cytoplasm, the thiol-disulfide oxidoreductase activity was expectedly nonessential. DnaJ and ΔDnaJ mutant that was deleted in the 55 residue cysteine-rich domain, both drove at the same maximal velocity the ATP-fuelled DnaK-driven unfolding/refolding of stable pre-aggregated polypeptide substrates. This confirmed earlier observations that under reducing conditions, natural type II and III J-proteins, such as CbpA, DjlA or human DNAJB1 and DNAJB4 can functionally replace type I J-proteins (Ueguchi et al., 1994; Bird et al., 2006; Hinault et al., 2010). Mutations in DnaJ have shown that although non-essential, the Zn-binding domain contributes in part to the binding of the protein substrate (Linke et al., 2003). Indeed, we found that the EC_50_value for ΔDnaJ was about three times higher than for DnaJ, suggesting that type II J-proteins in general may bind less tightly to their misfolded substrates (Figure 3B).

Under mildly oxidative conditions, we found that the cysteine-rich domain was central to the ability of the Hsp70 chaperone to act on partially oxidized misfolded substrates as an ATP-dependant unfolding machine and to successfully convert them into natively refolded proteins. The effective thiol-reductase activity of WT DnaJ on insulin under limited reducing conditions, which we found to be missing in the ΔDnaJ, was a strong evidence that in order to accelerate the transfer proton and electrons from DTT to the oxidized disulfide bonds of the misfolded rhodanese substrate, DnaJ must have been able to transiently form covalent bonds between its cysteine-rich domain and the target misfolded polypeptides.

In addition to thioredoxin-1, which has a unique WCGPC motif, *E. coli* also expresses under stress thioredoxin-2 that carries two additional CXXC pairs and binds zinc with a very high affinity. Thioredoxin-2 is as efficient as thioredoxin-1 at reducing disulfide bonds in proteins (Collet et al., 2003). This indicates that the tight Zinc binding by the CXXC motives of type 1 J-proteins is not incompatible with their apparent ability to carry a catalytic thiol-disulfide oxidoreductase activity. Possibly, the role of the two Zn ions is to merely stabilize the anti-parallel beta-sheet conformation of the cysteine-rich domain.

The presence of both type I and II J-proteins in the eukaryotic cytosol suggests that without stress, both type I and II may be nearly as effective co-chaperones of the Hsp70s and Hsp110s. Yet, under mild oxidative stress, type I J-proteins may provide a powerful synergism between their intrinsic thiol-disulfide oxidoreductase “foldase” type of activity and the ATP-fuelled polypeptide unfoldase activity of Hsp70s and Hsp110s.

How may a thiol-disulfide oxidoreductase act as a polypeptide foldase? When binding to a tensed misaligned disulfide bond in a misfolded polypeptide, the enzyme is expected to transiently open it. This may suffice to release structural tensions in the polypeptide which can then reach a more native conformation, which upon the thiol-disulfide oxidoreductase dissociation can either form a new stabilizing disulfide bond between properly aligned cysteine pairs, or necessitate the assisted reduction of the cysteines by NADPH-dependant or glutathione thioredoxin reductates (Creighton and Goldenberg, 1984; Schwaller et al., 2003). Our results showed that the cysteine rich domain conferred an intrinsic thiol-disulfide oxidoreductase “foldase” activity to DnaJ, which is lacking in ΔDnaJ and could be partially alleviated upon external addition of thioredoxin.

Thioredoxin-like proteins are essential to the folding of mildly oxidized proteins in cellular compartments that are often exposed to oxidative stress, such as the ER lumen or the chloroplast that accumulate ROS during photosynthesis. The Bundle Sheath Defective-2 (BSD2) is a 129-residue chloroplast imported protein, which is important for the proper assembly of the RubisCO holoenzyme (Brutnell et al., 1999). In chloroplast of angiosperms and gymnosperms (Supplementary Figure 2, upper panel), mature BSD2 is likely a 86 residues protein with four typical CXXC pairs that can form two Zn-fingers, as in the case of the cysteine rich domain of Type I J-proteins (Supplementary Figure 2, lower panel). Because it lacks a typical J-domain to bind Hsp70s, it is wrongly annotated as a member of the DnaJ/Hsp40 superfamily. Yet, its strong homology with the cysteine-rich domain of type I J-proteins suggests that when expressed in these cellular compartments, type II J-proteins that are less effective at chaperoning the Hsp70-mediated unfolding of partially oxidized misfolded proteins, might be synergized in trans by thiol-disulfide oxidoreductase, such as BSD2.

With some exceptions, proteins in the reducing environment of unstressed eukaryotic cytosol or bacterial cytoplasm have no disulfide bonds and they rarely align vicinal cysteines that could form a disulfide bond under oxidative stress without damaging the native structure (Kolberg et al., 2004; Saaranen and Ruddock, 2013). For example, in the bovine rhodanese structure, the two closest cysteine residues (C_248_, C_255_) don't face each other and are 7.5 Å apart, implying that the formation of 2.05 Å-long disulfide bonds under mild oxidative stress, would have to stabilize only severely distorted non-native conformation of the polypeptide. Under such conditions, the intrinsic thiol-disulfide oxidoreductase activity associated to the Type I J-proteins of the Hsp70 systems (DnaK-DnaJ-GrpE) in the cytosol, which by virtue of their ability to specifically bind to misfolded proteins rather than to natively folded or natively unfolded proteins (Hinault et al., 2010) would provide a great advantage to the cell's attempts to rescue misfolded mildly oxidized polypeptides, such as rhodanese, stabilized by wrong disulfide bonds.

Working with mildly oxidized misfolded rhodanese, which contains four cysteines, we observed that the DnaJ mutant lacking a thiol-oxidoreductase activity of its own, was less effective at mediating the DnaK-ATP-mediated unfolding/refolding reaction than wild type DnaJ that contain a thiol-oxidoreductase activity of its own (Figure 4A). This stongly suggests that a major limiting factor of the ATP-mediated unfolding/refolding reaction was the presence of wrong disulfide bonds in the substrate, rather than in the DnaK and DnaJ molecules, which needed to be transiently opened by the cysteine rich domain or by externally added thioredoxin, in order to be effectively unfolded and refolded to the native state.

**Figure 5:**
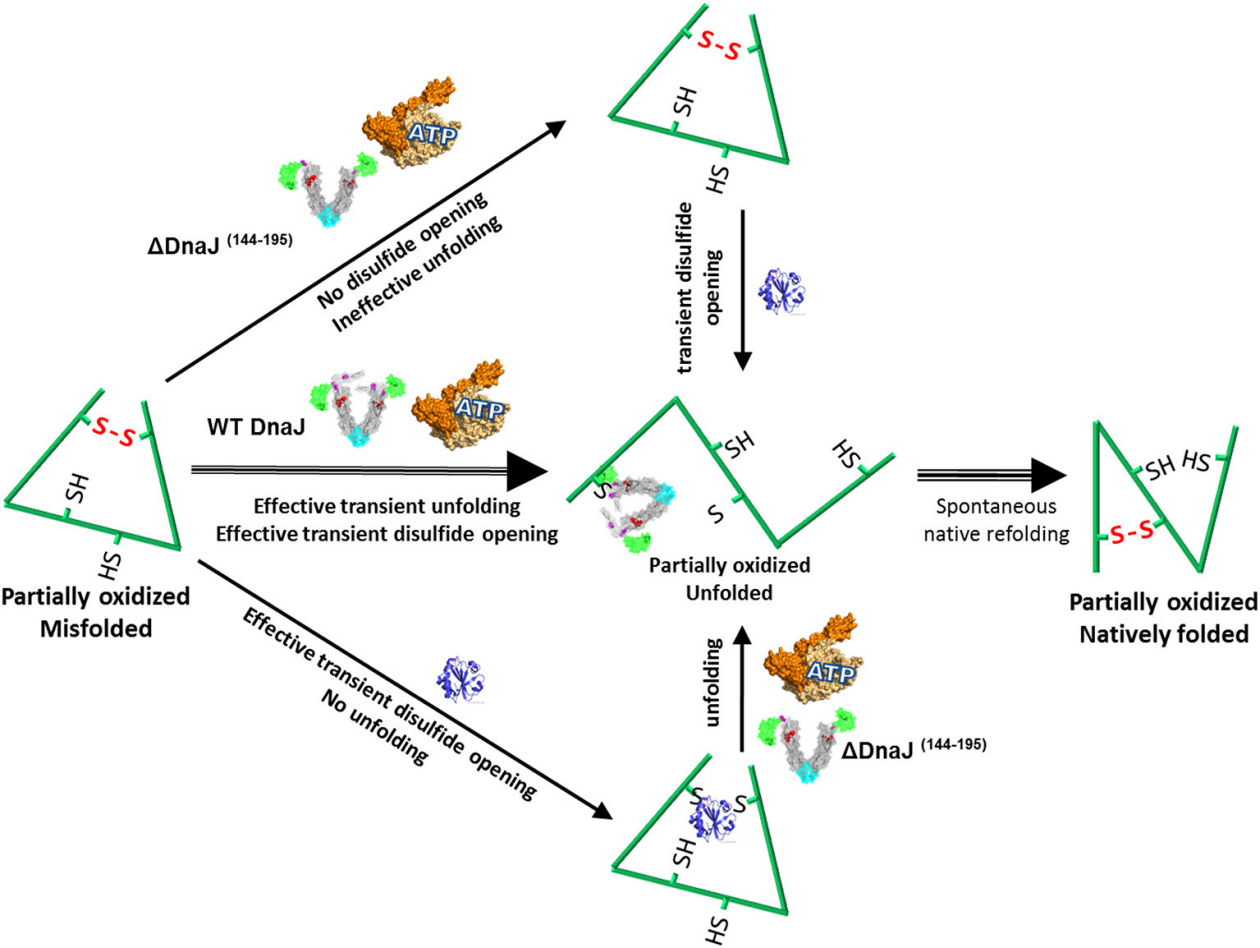
**Proposed model for the concerted dithiol-oxidoreductase and unfoldase activities by type I J-co-chaperones**. Under mildly oxidative conditions, a stress-misfolded polypeptide may be stabilized both by misaligned disulfide bonds and by strong cooperative hydrophobic interactions, for example in wrong beta sheets. Wild type DnaJ can recruit DnaK to forcefully unfold wrong beta structures and at the same time catalyze the transient opening of wrong disulfide bonds. Thus a partially unfolded intermediate is produced, which can thereafter spontaneously refold to the native state (Strong middle arrows). In the presence of the ΔDnaJ, wrong disulfide bonds cannot be opened and DnaK remains ineffective at unfolding the wrong structures, unless thioredoxin is subsequently added (upper arrows). In the presence of thioredoxin, wrong disulfide bonds are transiently untied but there is no unfolding of wrong beta structures, unless ΔDnaJ, DnaK and ATP are subsequently added (lower arrows).

Together, our data suggest that in case the mere transient opening of a wrong disulfide bond by the inbuilt thiol-disulfide oxidoreductase domain of the Type I J-protein, does not suffice to disentangle a polypeptide from its severely distorted conformation (Figure 5, lower path), the bound co-chaperone can also recruit the Hsp70 chaperone machinery, which upon ATP hydrolysis can unfold the “frozen” misfolded segment, thereby allowing it, upon release, to refold to the native state (Figure 5, middle path). In the specific case of type II J-co-chaperones (or ΔDnaJ), the unfolding action of DnaK may remain futile (Figure 5, upper path), unless a thioredoxin is supplemented.

Yet, under more extreme oxidative, ΔDnaJ of Type 2 J-proteins could also be slightly more effective J-cochaperones than Type 1 at mediating the unfolding of particularly sensitive proteins, such as heat-denatured G6PDH. This may be attributed to the presence of the eight reduced cysteines that can form dysfunctional disulfide bonds with the other proteins. Hence, under oxidative stress, the more resistant type II J-proteins, such as the *E. coli* CbpA or the human DNAJB1, may be more effective at assisting protein unfolding, than the oxygen-sensitive type I DnaJ or DNAJA1. Excessive sensitivity of type I J-proteins to oxidative conditions could explain their absence in the oxidative environment of the ER lumen and the presence of only cysteine-depleted type II and III J-proteins.

Protein misfolding and aggregation likely appeared in the course of evolution when small single domain proteins became complex and multi-domain (Netzer and Hartl, 1997), implying that the first molecular chaperone, such a DnaK and DnaJ, likely evolved under a reducing atmosphere devoid of free oxygen (Gupta et al., 1989; Picketts et al., 1989; Ellis and van der Vies, 1991). It is tempting to speculate that in the earliest anoxic life forms, the first J-proteins were simple type II and only later, a BSD2-like thiol-disulfide oxidoreductase modular domain was added to form type I J-proteins to optimize the unfoldase action of Hsp70 under mild oxygen.

### The possible function of type I and II in protein misfolding diseases

We performed a Genevestigator analysis of mRNA expression levels in human cells (Zimmermann et al., 2004) challenged by various stresses associated to oxidative stress and ROS production. Thus heat-shock, cigarette smoke, or chemical treatments with elesclomol or the HSP90-inhibitor geldanamycin, as well as in chronically challenged tissues in Parkinson's disease, type II, DNAJB1 and DNAJB4 were found to be systematically more over-expressed than type I, DNAJA1 and DNAJA2 (Supplementary Figure 3). This is possibly because extreme persistent oxidative and inflammation stress, as in cells of the *substantia nigra* during progression of Parkinson's disease, which are chronically challenged with toxic aggregates that compromise membranes integrity, the excessive ROS production might inactivate and crosslink the highly reactive catalytic cysteines in type I J-proteins and cause inactivation by intra- and inter-molecular crosslinks. Under such extreme oxidative conditions, it would be counter-productive for cells to overproduce type I co-chaperones and more advantageous to favor the production of ROS-resistant although less performing type-II cochaperones and possibly in collaboration with more appropriate Thioredoxins, such as BSD2, to effectively unfold otherwise oxidized misfolded polypeptides. Our results suggest that the specific upregulation in aging cells of type I J-proteins or Thioredoxins, in combination with Hsp110 and Hsc70 (Mattoo et al., 2013) could be of important therapeutic potential to combat ROS-mediated stress and inflammation-induced onset of proteotoxicity in degenerative diseases.

### Conflict of interest statement

The authors declare that the research was conducted in the absence of any commercial or financial relationships that could be construed as a potential conflict of interest.
