# Supplementary material for: Synergism between a foldase and an unfoldase: reciprocal dependence between the thioredoxin-like activity of DnaJ and the polypeptide-unfolding activity of DnaK
Source: Front Mol Biosci. 2014 Jul 31;1:7. doi: 10.3389/fmolb.2014.00007 (PMC4428491; doi:10.3389/fmolb.2014.00007)
Supplement: Supplementary file 1 [file DataSheet1.PDF]

8 *Supplementary Material*

Synergism between a foldase and an unfoldase: Reciprocal dependence between the thioredoxin-like activity of DnaJ and the polypeptide-unfolding activity of DnaK

Rayees U.H. Mattoo[1], America Farina Henriquez Cuendet[1], Sujatha Subanna[1], Andrija Finka[1], Smriti Priya[1], Sandeep K. Sharma[1] and Pierre Goloubinoff[1]*

[1]DBMV, Faculty of Biology and Medicine, Biophore building, University of Lausanne, 1015-Lausanne, Switzerland.

*To whom correspondence should be addressed: Pierre.Goloubinoff@unil.ch

Supplementary Figure 1

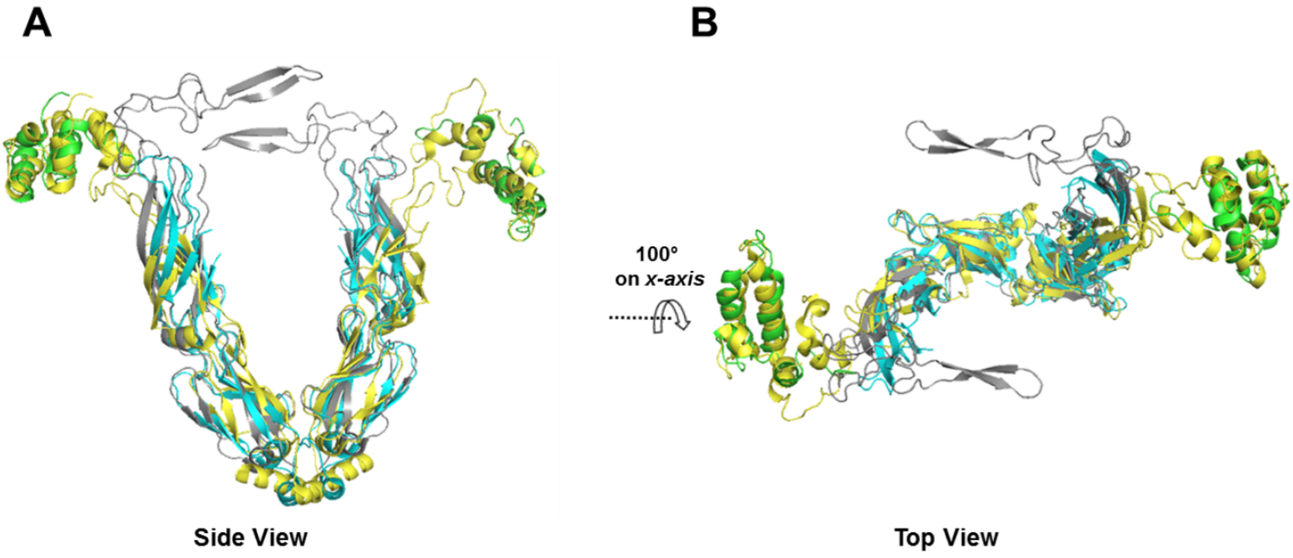

Supplementary Figure 1: Structure reconstitution of an *E. coli* DnaJ dimer. For the approximate three-dimensional reconstitution of a typical DnaJ dimmer, we used several partial structures from X-ray crystallography and NMR of yeast Hsp40, bacterial CbpA and DnaJ: Hsp40, ydj1 (PDB: 1NLT) consisted of cysteine-rich region and part of C-terminal domain but lacking J-domain and dimerization region, *gray*. The structure of *Thermus thermophilus* DnaJ (PDB: 4J80), which is cysteine-less CbpA type II J-protein, consisted of a J-domain and a near complete protein-binding C-terminal protein-binding domain with a dimerization region, *yellow*. The crystal structure of human Hsp40 (hdj1) (PDB: 3AGY) consisted of a near complete C-terminal protein-binding domain but was lacking the J-domain and cysteine rich region, *cyan*. The structure of the J-domain alone was from *E. coli* DnaJ (PDB: 1BQ0), *green*.

Supplementary Figure 2

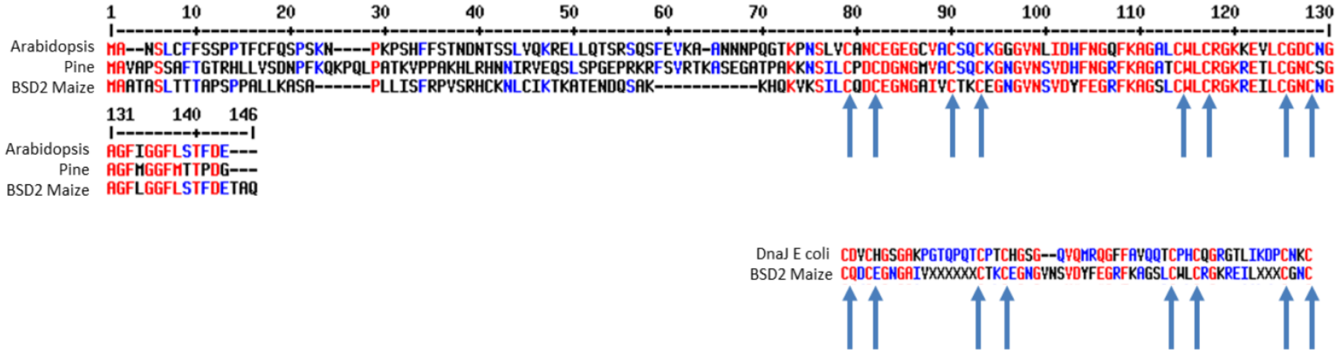

Supplementary Figure 2: Sequence alignment of Bundle Sheath Defective-2 (BSD2) with J-proteins. *Upper panel:* Sequence alignment of chloroplast BSD2 from maize, with the BSD2 orthologs in *Arabidopsis* (AT3G47650) and Pine tree (*Picea sitchensis* ABK21437). Following a variable chloroplast import segment, there is a conserved Zinc-binding domain with four typical CXXC pairs as in DnaJ but without the J domain and the protein-binding domain. *Lower panel*: sequence alignment of the Zinc-binding domain of maize BSD2 and *E. coli* DnaJ. The cysteine pairs are indicated with double arrows.

Supplementary Figure 3

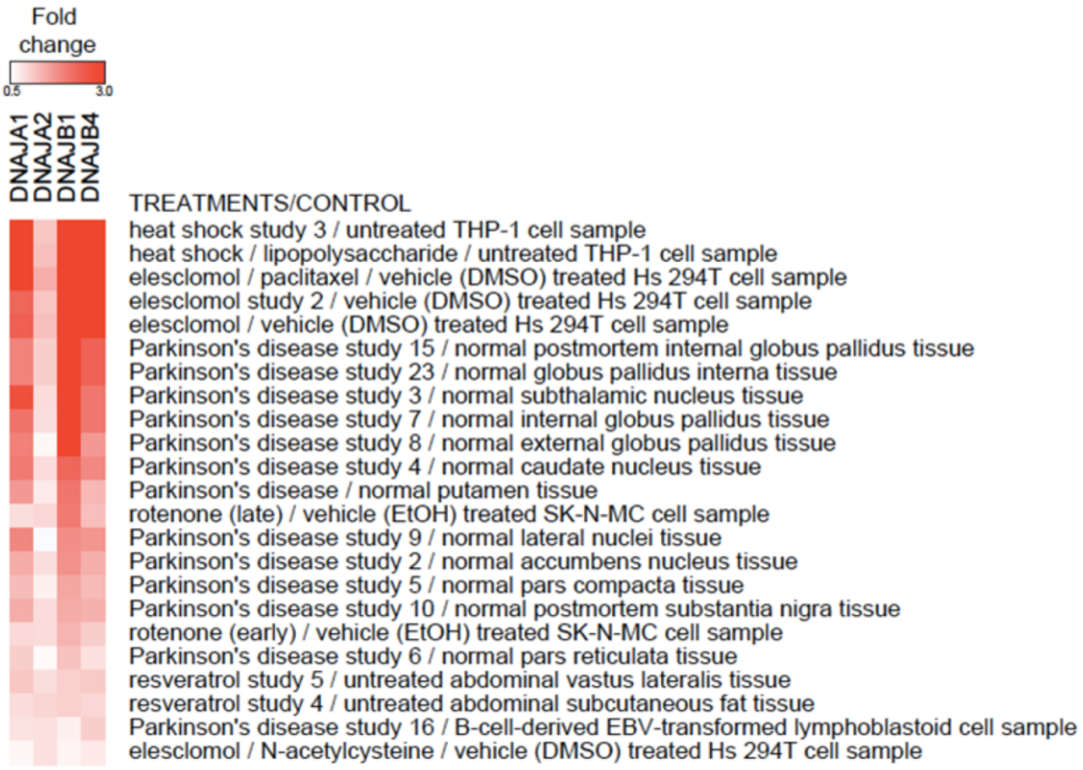

Supplementary Figure 3. Various stresses induce less sensitive type I than resistant type II J-domain proteins. Genevestigator analysis of relative mRNA expression levels of the major human cytoplasmic J-domain proteins type I (DNAJA1 and DNAJA2) containing a cysteine-rich domain and type II (DNAJB1 and DNAJB4) lacking a cysteine-rich domain, in various immortal human cell lines, following heat- or chemical treatments, or in constantly distressed degenerative brain tissues in Parkinson disease. The relative mRNA fold increase levels of DNAJA1, DNAJA2 and DNAJB4 were arranged according to the descending values of DNAJB1.
